# Supplementary figures and images for: Identification and validation of prognostic and tumor microenvironment characteristics of necroptosis index and BIRC3 in clear cell renal cell carcinoma
Source: PeerJ. 2023 Dec 18;11:e16643. doi: 10.7717/peerj.16643 (PMC10734432; doi:10.7717/peerj.16643)

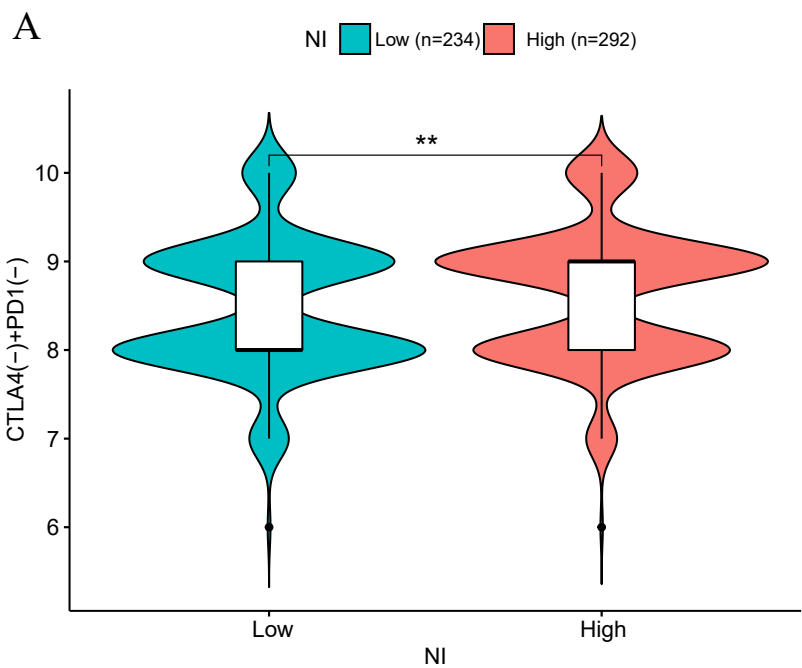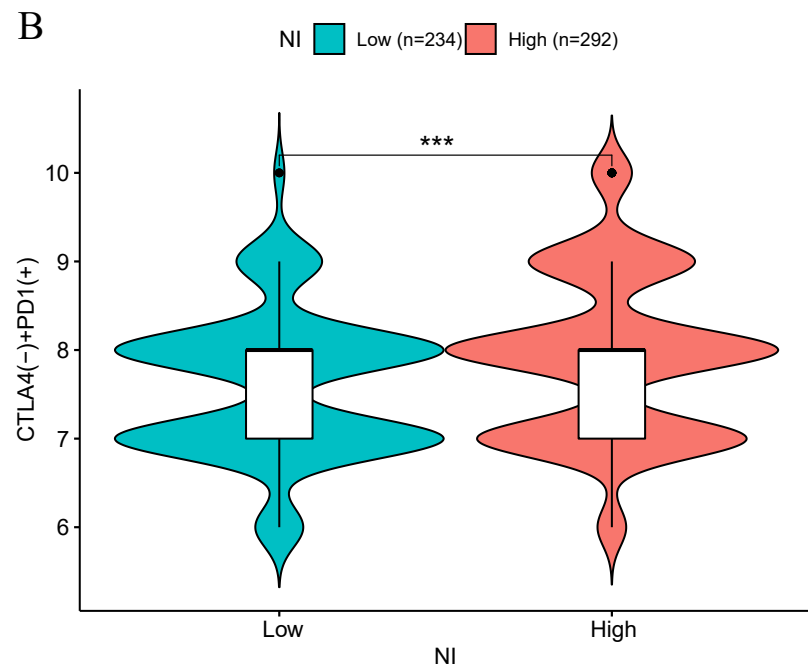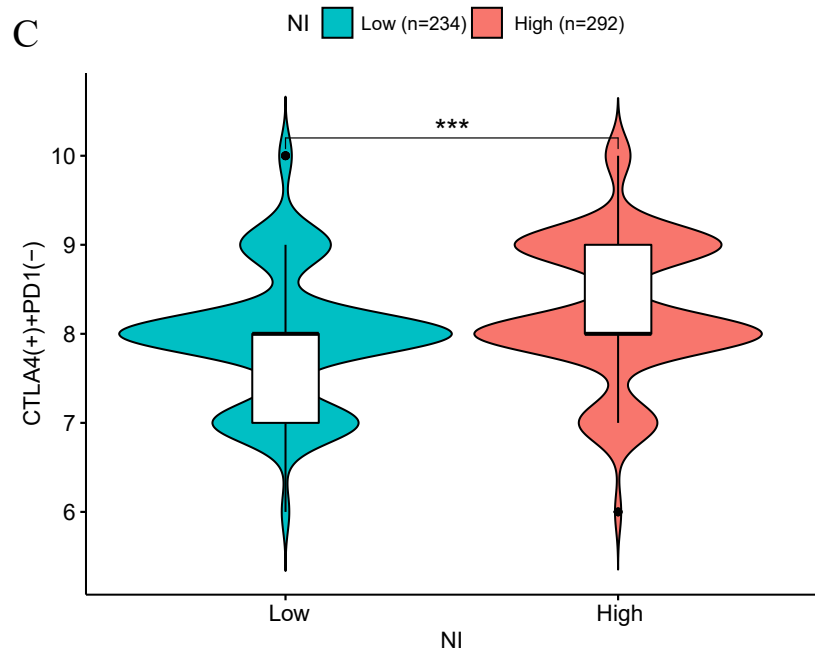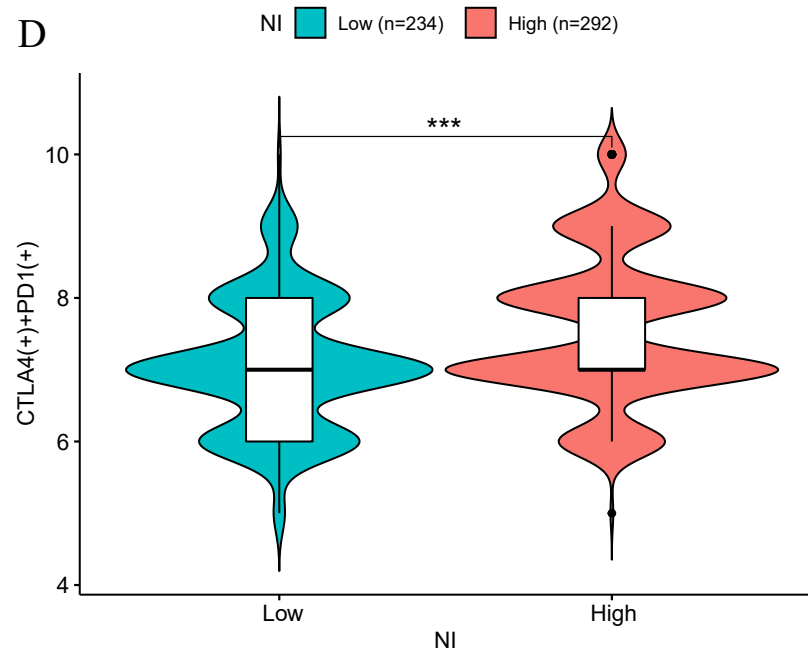

Figure S1. The immunotherapy score differences in high and low NI groups

Supplement: Supplemental Information 1 — The violin plots of immunethrapy score of low- and high-NI groups. (A) CTLA4(−)+PD1(−), 95% CI: [0.03095–0.2875]; (B) CTLA4(−)+PD1(+), 95% CI: [0.1514 to 0.4516]; (C) CTLA4(+)+PD1(−), 95% CI: [0.2203–0.4815]; (D) CTLA4(+) +PD1(+); High NI group: 292, Low NI group: 234. [file peerj-11-16643-s001.pdf]

## BIRC3-2

200kDa-  
140kDa-  
110kDa-  
75kDa-  
55kDa-  
42kDa-  
30kDa-  
23kDa-  
18kDa-

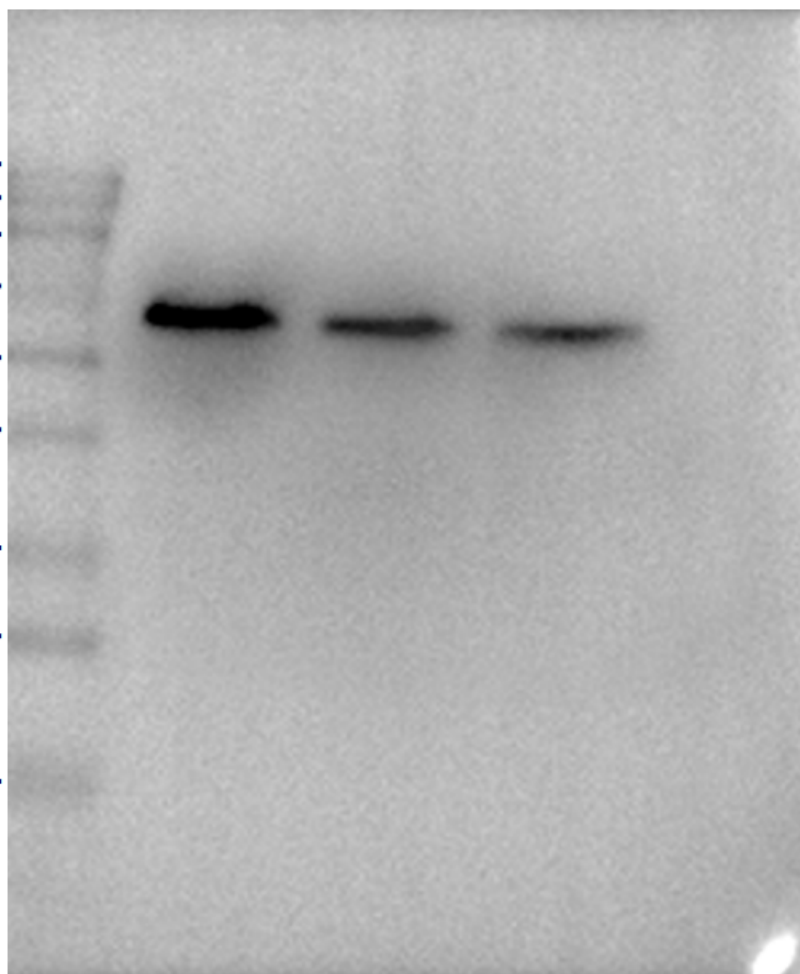

Supplement: Supplemental Information 7 [file peerj-11-16643-s007.pdf]

# GAPDH-1

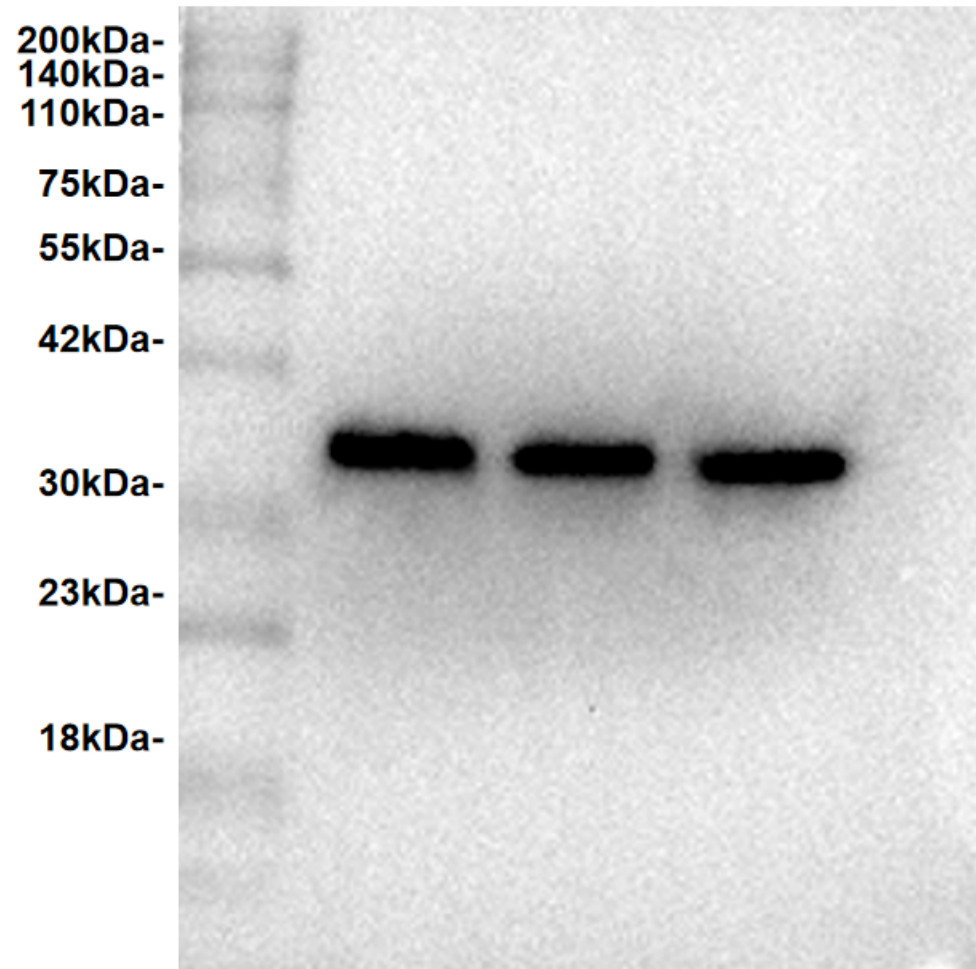

Supplement: Supplemental Information 8 [file peerj-11-16643-s008.pdf]

# BIRC3-1

200kDa-  
140kDa-  
110kDa-  
  
75kDa-  
55kDa-  
  
42kDa-  
  
30kDa-  
  
23kDa-  
  
18kDa-

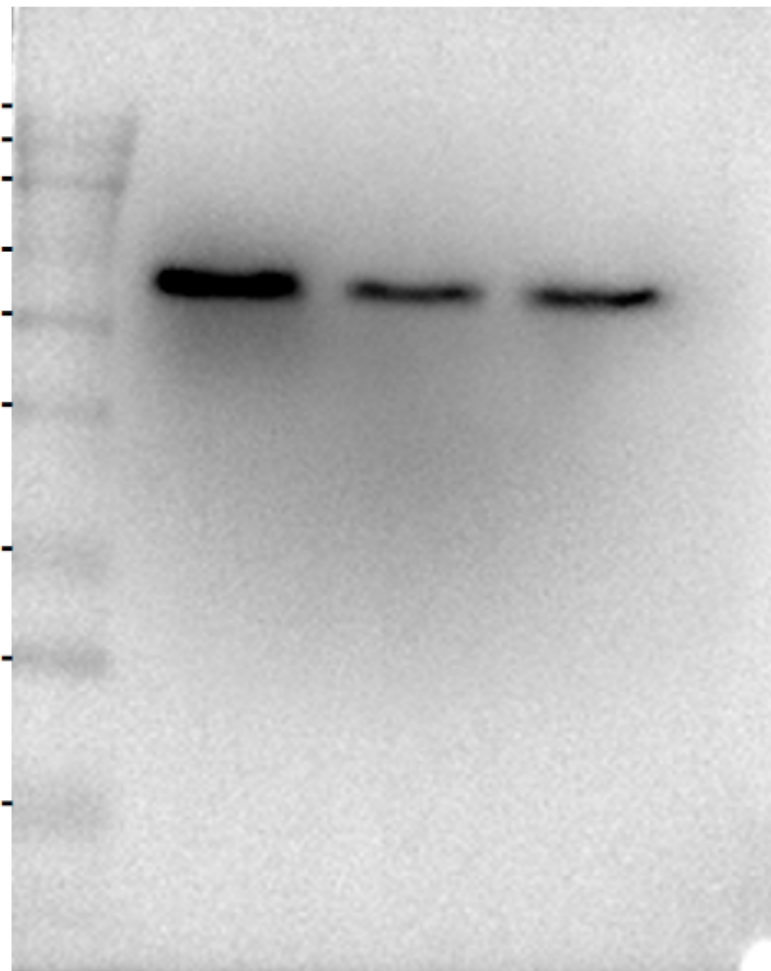

Supplement: Supplemental Information 9 [file peerj-11-16643-s009.pdf]

## GAPDH-2

200kDa-  
140kDa-  
110kDa-  
  
75kDa-  
55kDa-  
  
42kDa-  
  
30kDa-  
  
23kDa-  
  
18kDa-

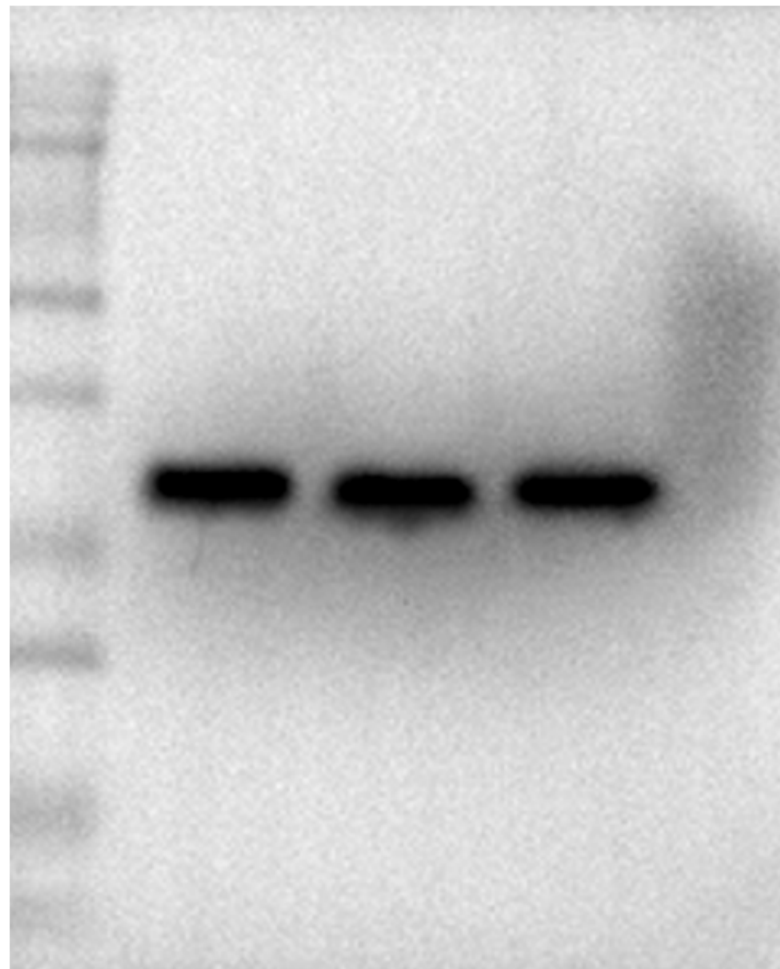

Supplement: Supplemental Information 10 [file peerj-11-16643-s010.pdf]
