# Supplementary material for: Identification and validation of prognostic and tumor microenvironment characteristics of necroptosis index and BIRC3 in clear cell renal cell carcinoma
Source: PeerJ. 2023 Dec 18;11:e16643. doi: 10.7717/peerj.16643 (PMC10734432; doi:10.7717/peerj.16643)
Supplement: Supplemental Information 2 — (A) Differential expression of NI in different clinicopathological characteristics (A: cluster; B: Grade; C: Stage; D: T; E: M; F: N). (G–L)The OS Kaplan-Meier curve of NI in different clinicopathological characteristics. [file peerj-11-16643-s002.pdf]

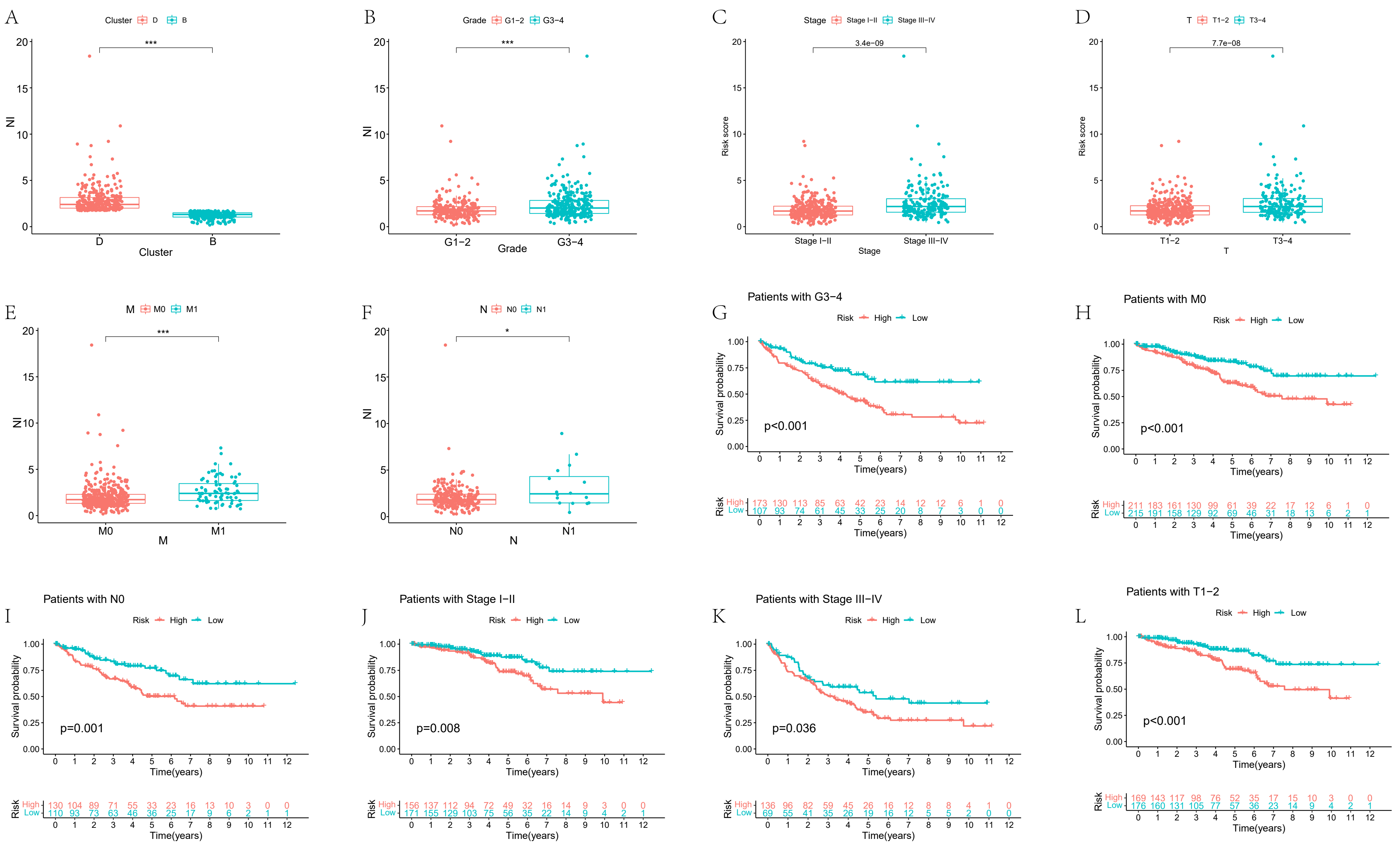

Figure S2. Validation of the prognosis of NI in the different clinicopathological characteristics.
