# Supplementary material for: Identification and validation of prognostic and tumor microenvironment characteristics of necroptosis index and BIRC3 in clear cell renal cell carcinoma
Source: PeerJ. 2023 Dec 18;11:e16643. doi: 10.7717/peerj.16643 (PMC10734432; doi:10.7717/peerj.16643)
Supplement: Supplemental Information 3 — <!--[if !supportLists]-->(A)<!--[endif]-->Grade; (B) Stage; (C) T; (D) M; (E) N; (F) ROC of six modeled NRGs; <!--[if !supportLists]-->(G)<!--[endif]-->Correlation between genes and immune cells; (H) Correlation between genes and immune checkpoints. [file peerj-11-16643-s003.pdf]

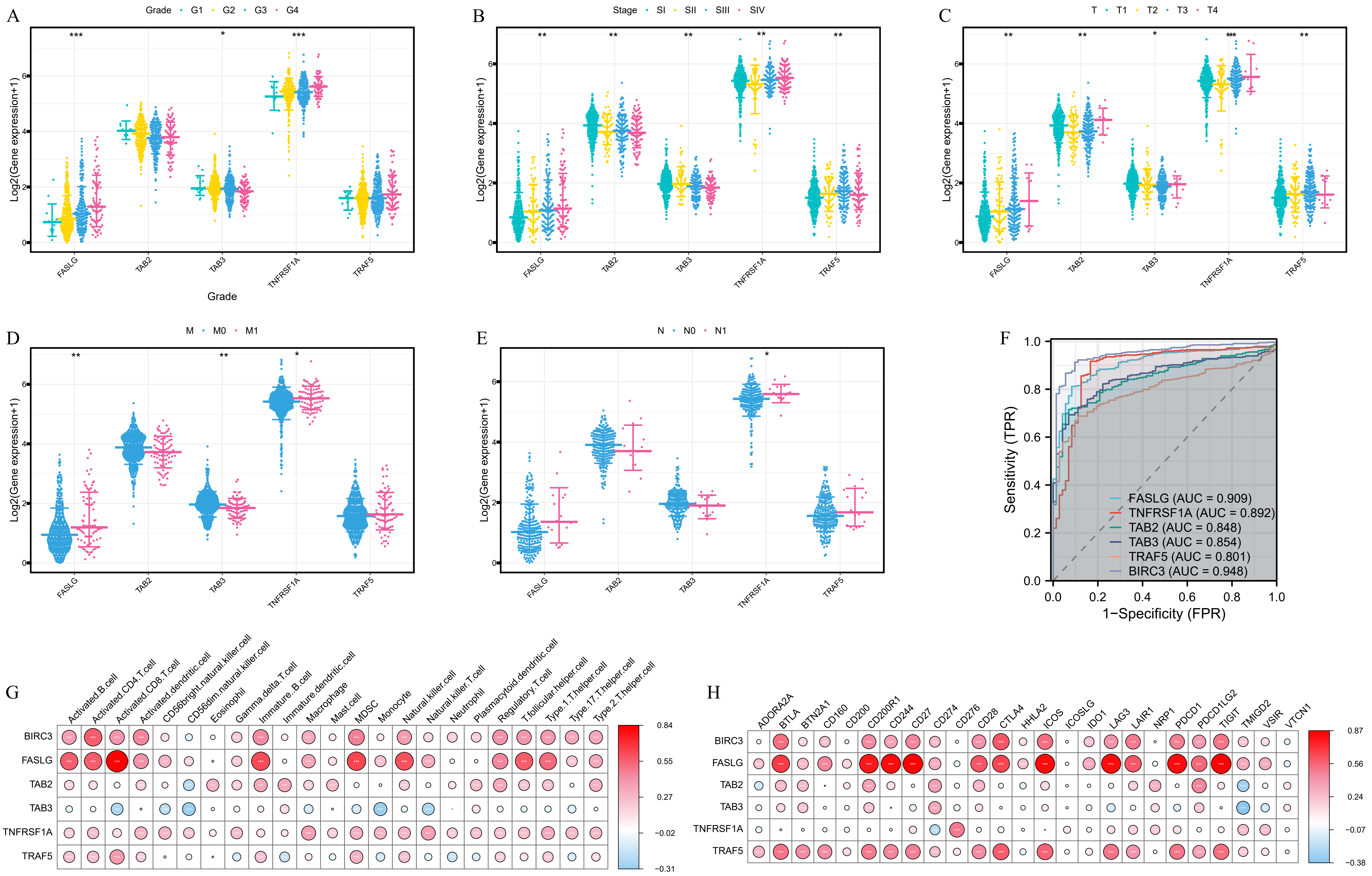

Figure S3. Different expression of prognostic genes among clinical pathological and immune infiltration characteristics.
(A)Grade; (B) Stage; (C) T; (D) M; (E) N; (F) ROC of six modeled NRGs;
(G) Correlation between genes and immune cells; (H) Correlation between genes and immune checkpoints
