# Supplementary material for: Identification and validation of prognostic and tumor microenvironment characteristics of necroptosis index and BIRC3 in clear cell renal cell carcinoma
Source: PeerJ. 2023 Dec 18;11:e16643. doi: 10.7717/peerj.16643 (PMC10734432; doi:10.7717/peerj.16643)
Supplement: Supplemental Information 5 [file peerj-11-16643-s005.docx]

| **Clinical features** | **TCGA**  **(Tumor=539**  **Normal=72)** | | **GSE73731**  **(Tumor=265**  **Normal=0)** | | **GSE53737 (Tumor=72**  **Normal=72)** | | **GSE17895**  **(Tumor=138**  **Normal=22)** | | **GSE36895 (Tumor=29**  **Normal=23)** | | **GSE66272 (Tumor=27**  **Normal=27)** | |
| --- | --- | --- | --- | --- | --- | --- | --- | --- | --- | --- | --- | --- |
|  | **N** | **%** | **N** | **%** | **N** | **%** | **N** | **%** | **N** | **%** | **N** | **%** |
| Age (years) |  |  | - |  | - |  | - |  | - |  | - |  |
| ≤65 | 353 | 65.49 |  |  |  |  |  |  |  |  |  |  |
| >65 | 186 | 34.51 |  |  |  |  |  |  |  |  |  |  |
| Gender |  |  |  |  | - |  | - |  | - |  | - |  |
| Female | 186 | 34.51 |  |  |  |  |  |  |  |  |  |  |
| Male | 353 | 65.49 |  |  |  |  |  |  |  |  |  |  |
| Grade |  |  |  |  | - |  | - |  | - |  | - |  |
| G1 | 14 | 2.60 | 22 | 8.30 |  |  |  |  |  |  |  |  |
| G2 | 235 | 43.60 | 90 | 33.96 |  |  |  |  |  |  |  |  |
| G3 | 207 | 38.40 | 95 | 35.85 |  |  |  |  |  |  |  |  |
| G4 | 75 | 13.91 | 49 | 18.49 |  |  |  |  |  |  |  |  |
| Gx | 5 | 0.93 | - | - |  |  |  |  |  |  |  |  |
| Unknown | 3 | 0.56 | 9 | 3.40 |  |  |  |  |  |  |  |  |
| T stage |  |  | - |  | - |  | - |  | - |  | - |  |
| T1 | 278 | 51.58 |  |  |  |  |  |  |  |  |  |  |
| T2 | 71 | 13.17 |  |  |  |  |  |  |  |  |  |  |
| T3 | 179 | 33.21 |  |  |  |  |  |  |  |  |  |  |
| T4 | 11 | 2.04 |  |  |  |  |  |  |  |  |  |  |
| N stage |  |  | - |  | - |  | - |  | - |  | - |  |
| N0 | 241 | 44.71 |  |  |  |  |  |  |  |  |  |  |
| N1 | 16 | 2.97 |  |  |  |  |  |  |  |  |  |  |
| NX | 282 | 52.32 |  |  |  |  |  |  |  |  |  |  |
| M stage |  |  | - |  | - |  | - |  | - |  | - |  |
| M0 | 428 | 79.41 |  |  |  |  |  |  |  |  |  |  |
| M1 | 78 | 14.47 |  |  |  |  |  |  |  |  |  |  |
| MX | 31 | 5.75 |  |  |  |  |  |  |  |  |  |  |
| Unknown | 2 | 0.37 |  |  |  |  |  |  |  |  |  |  |
| Stage |  |  |  |  |  |  | - |  | - |  | - |  |
| Stage I | 272 | 50.46 | 41 | 15.47 | 24 | 33.33 |  |  |  |  |  |  |
| Stage II | 59 | 10.95 | 12 | 4.53 | 14 | 19.44 |  |  |  |  |  |  |
| Stage III | 123 | 22.82 | 28 | 10.57 | 19 | 26.39 |  |  |  |  |  |  |
| Stage IV | 82 | 15.21 | 44 | 16.60 | 15 | 20.83 |  |  |  |  |  |  |
| Unknown | 3 | 0.56 | 140 | 52.83 | - | - |  |  |  |  |  |  |

Table S1. Clinical characteristics of clear cell renal cell carcinoma patients in multiple databases
