# Supplementary material for: Identification and validation of prognostic and tumor microenvironment characteristics of necroptosis index and BIRC3 in clear cell renal cell carcinoma
Source: PeerJ. 2023 Dec 18;11:e16643. doi: 10.7717/peerj.16643 (PMC10734432; doi:10.7717/peerj.16643)
Supplement: Supplemental Information 6 [file peerj-11-16643-s006.docx]

Table S2. The scoring data of ccRCC immunotherapy cases.

| ID | CTLA4(-)+  PD1(-) | CTLA4(-)+  PD1(+) | CTLA4(+)+  PD1(-) | CTLA4(+)+  PD1(+) | Risk |
| --- | --- | --- | --- | --- | --- |
| TCGA-3Z-A93Z | 9 | 8 | 9 | 7 | High |
| TCGA-6D-AA2E | 8 | 8 | 7 | 8 | Low |
| TCGA-A3-3306 | 8 | 7 | 8 | 7 | High |
| TCGA-A3-3307 | 9 | 8 | 8 | 8 | High |
| TCGA-A3-3308 | 8 | 7 | 7 | 7 | High |
| TCGA-A3-3311 | 9 | 8 | 8 | 8 | Low |
| TCGA-A3-3313 | 7 | 6 | 6 | 6 | Low |
| TCGA-A3-3316 | 8 | 7 | 7 | 6 | High |
| TCGA-A3-3317 | 9 | 8 | 8 | 7 | High |
| TCGA-A3-3319 | 8 | 7 | 8 | 6 | High |
| TCGA-A3-3320 | 8 | 7 | 7 | 6 | Low |
| TCGA-A3-3322 | 8 | 7 | 7 | 6 | Low |
| TCGA-A3-3323 | 8 | 8 | 8 | 7 | Low |
| TCGA-A3-3324 | 8 | 8 | 7 | 7 | Low |
| TCGA-A3-3325 | 8 | 7 | 8 | 7 | High |
| TCGA-A3-3326 | 9 | 9 | 9 | 8 | Low |
| TCGA-A3-3328 | 9 | 8 | 8 | 7 | Low |
| TCGA-A3-3329 | 9 | 8 | 8 | 8 | Low |
| TCGA-A3-3331 | 8 | 7 | 7 | 7 | Low |
| TCGA-A3-3335 | 8 | 7 | 8 | 7 | High |
| TCGA-A3-3343 | 9 | 8 | 8 | 8 | Low |
| TCGA-A3-3346 | 8 | 7 | 7 | 7 | High |
| TCGA-A3-3347 | 8 | 7 | 7 | 7 | High |
| TCGA-A3-3349 | 8 | 7 | 8 | 6 | High |
| TCGA-A3-3351 | 9 | 9 | 9 | 9 | High |
| TCGA-A3-3352 | 8 | 7 | 8 | 7 | Low |
| TCGA-A3-3357 | 10 | 9 | 9 | 9 | Low |
| TCGA-A3-3358 | 8 | 8 | 8 | 7 | Low |
| TCGA-A3-3359 | 8 | 8 | 8 | 8 | High |
| TCGA-A3-3362 | 8 | 7 | 8 | 7 | Low |
| TCGA-A3-3363 | 8 | 7 | 8 | 6 | Low |
| TCGA-A3-3365 | 9 | 8 | 9 | 7 | Low |
| TCGA-A3-3367 | 8 | 7 | 8 | 7 | Low |
| TCGA-A3-3370 | 9 | 9 | 9 | 9 | Low |
| TCGA-A3-3372 | 8 | 8 | 8 | 7 | Low |
| TCGA-A3-3373 | 8 | 7 | 8 | 7 | Low |
| TCGA-A3-3374 | 7 | 7 | 7 | 7 | Low |
| TCGA-A3-3376 | 8 | 7 | 8 | 7 | Low |
| TCGA-A3-3378 | 7 | 8 | 7 | 8 | High |
| TCGA-A3-3380 | 8 | 7 | 8 | 7 | High |
| TCGA-A3-3382 | 8 | 7 | 7 | 7 | Low |
| TCGA-A3-3383 | 9 | 8 | 9 | 8 | High |
| TCGA-A3-3385 | 8 | 7 | 7 | 6 | Low |
| TCGA-A3-3387 | 8 | 7 | 8 | 7 | Low |
| TCGA-A3-A6NI | 8 | 7 | 8 | 7 | High |
| TCGA-A3-A6NJ | 9 | 9 | 9 | 9 | High |
| TCGA-A3-A6NL | 8 | 7 | 8 | 7 | High |
| TCGA-A3-A6NN | 8 | 8 | 8 | 7 | High |
| TCGA-A3-A8CQ | 9 | 7 | 8 | 7 | High |
| TCGA-A3-A8OV | 9 | 8 | 9 | 7 | High |
| TCGA-A3-A8OW | 9 | 9 | 9 | 8 | High |
| TCGA-AK-3425 | 8 | 8 | 8 | 7 | High |
| TCGA-AK-3426 | 8 | 8 | 8 | 8 | High |
| TCGA-AK-3427 | 10 | 8 | 9 | 7 | Low |
| TCGA-AK-3428 | 8 | 7 | 7 | 6 | High |
| TCGA-AK-3429 | 9 | 9 | 9 | 9 | High |
| TCGA-AK-3431 | 8 | 7 | 8 | 6 | High |
| TCGA-AK-3433 | 9 | 7 | 8 | 7 | Low |
| TCGA-AK-3434 | 10 | 9 | 10 | 9 | High |
| TCGA-AK-3436 | 9 | 7 | 8 | 7 | High |
| TCGA-AK-3440 | 9 | 8 | 8 | 7 | Low |
| TCGA-AK-3443 | 10 | 9 | 10 | 9 | Low |
| TCGA-AK-3445 | 10 | 9 | 9 | 8 | High |
| TCGA-AK-3447 | 9 | 8 | 9 | 8 | Low |
| TCGA-AK-3450 | 8 | 7 | 8 | 6 | Low |
| TCGA-AK-3451 | 10 | 9 | 9 | 8 | Low |
| TCGA-AK-3453 | 8 | 7 | 7 | 6 | High |
| TCGA-AK-3454 | 9 | 8 | 8 | 7 | High |
| TCGA-AK-3455 | 10 | 10 | 10 | 10 | Low |
| TCGA-AK-3456 | 7 | 7 | 7 | 6 | Low |
| TCGA-AK-3458 | 10 | 9 | 10 | 8 | High |
| TCGA-AK-3460 | 9 | 8 | 8 | 7 | High |
| TCGA-AK-3461 | 9 | 8 | 8 | 8 | High |
| TCGA-AK-3465 | 9 | 7 | 8 | 7 | Low |
| TCGA-AS-3777 | 9 | 8 | 9 | 7 | Low |
| TCGA-AS-3778 | 8 | 7 | 7 | 6 | High |
| TCGA-B0-4688 | 6 | 7 | 6 | 6 | High |
| TCGA-B0-4690 | 7 | 7 | 7 | 7 | High |
| TCGA-B0-4691 | 10 | 9 | 9 | 8 | High |
| TCGA-B0-4693 | 8 | 8 | 8 | 7 | High |
| TCGA-B0-4694 | 8 | 8 | 8 | 7 | High |
| TCGA-B0-4696 | 8 | 6 | 7 | 5 | High |
| TCGA-B0-4697 | 9 | 9 | 9 | 10 | High |
| TCGA-B0-4698 | 7 | 8 | 7 | 8 | High |
| TCGA-B0-4699 | 7 | 8 | 7 | 8 | Low |
| TCGA-B0-4700 | 8 | 8 | 8 | 8 | High |
| TCGA-B0-4701 | 8 | 8 | 8 | 8 | High |
| TCGA-B0-4703 | 9 | 8 | 9 | 8 | High |
| TCGA-B0-4706 | 9 | 8 | 8 | 7 | High |
| TCGA-B0-4707 | 9 | 8 | 8 | 7 | High |
| TCGA-B0-4710 | 8 | 6 | 7 | 6 | High |
| TCGA-B0-4712 | 8 | 8 | 8 | 7 | High |
| TCGA-B0-4713 | 10 | 9 | 10 | 8 | High |
| TCGA-B0-4714 | 9 | 9 | 9 | 9 | High |
| TCGA-B0-4718 | 9 | 8 | 9 | 8 | High |
| TCGA-B0-4810 | 8 | 8 | 8 | 8 | High |
| TCGA-B0-4811 | 9 | 7 | 9 | 7 | High |
| TCGA-B0-4813 | 8 | 7 | 8 | 6 | High |
| TCGA-B0-4814 | 9 | 8 | 8 | 7 | Low |
| TCGA-B0-4815 | 9 | 9 | 8 | 9 | High |
| TCGA-B0-4816 | 8 | 7 | 8 | 7 | High |
| TCGA-B0-4817 | 9 | 8 | 9 | 8 | High |
| TCGA-B0-4818 | 9 | 9 | 9 | 8 | High |
| TCGA-B0-4819 | 8 | 8 | 9 | 9 | High |
| TCGA-B0-4821 | 8 | 7 | 8 | 7 | High |
| TCGA-B0-4822 | 8 | 6 | 7 | 6 | High |
| TCGA-B0-4823 | 9 | 8 | 8 | 7 | High |
| TCGA-B0-4824 | 9 | 9 | 9 | 9 | High |
| TCGA-B0-4827 | 8 | 8 | 8 | 7 | High |
| TCGA-B0-4828 | 9 | 8 | 9 | 7 | High |
| TCGA-B0-4833 | 9 | 8 | 9 | 8 | High |
| TCGA-B0-4834 | 9 | 8 | 8 | 7 | Low |
| TCGA-B0-4836 | 8 | 7 | 8 | 7 | High |
| TCGA-B0-4837 | 9 | 7 | 8 | 7 | High |
| TCGA-B0-4838 | 9 | 9 | 9 | 9 | High |
| TCGA-B0-4839 | 9 | 7 | 8 | 7 | High |
| TCGA-B0-4841 | 9 | 7 | 8 | 7 | High |
| TCGA-B0-4842 | 9 | 8 | 8 | 7 | High |
| TCGA-B0-4843 | 9 | 7 | 8 | 7 | High |
| TCGA-B0-4844 | 9 | 8 | 9 | 8 | High |
| TCGA-B0-4845 | 8 | 7 | 8 | 7 | High |
| TCGA-B0-4846 | 9 | 9 | 9 | 9 | High |
| TCGA-B0-4847 | 10 | 9 | 10 | 10 | High |
| TCGA-B0-4848 | 8 | 7 | 8 | 7 | High |
| TCGA-B0-4849 | 9 | 8 | 9 | 8 | High |
| TCGA-B0-4852 | 8 | 7 | 8 | 7 | Low |
| TCGA-B0-4945 | 9 | 8 | 9 | 8 | Low |
| TCGA-B0-5075 | 8 | 8 | 8 | 7 | Low |
| TCGA-B0-5077 | 10 | 8 | 9 | 8 | Low |
| TCGA-B0-5080 | 9 | 8 | 8 | 7 | High |
| TCGA-B0-5081 | 9 | 9 | 9 | 9 | High |
| TCGA-B0-5083 | 8 | 7 | 8 | 7 | Low |
| TCGA-B0-5084 | 8 | 7 | 8 | 6 | High |
| TCGA-B0-5085 | 9 | 8 | 9 | 7 | High |
| TCGA-B0-5088 | 9 | 8 | 8 | 7 | High |
| TCGA-B0-5092 | 10 | 10 | 10 | 10 | High |
| TCGA-B0-5094 | 8 | 7 | 8 | 6 | High |
| TCGA-B0-5095 | 8 | 7 | 8 | 7 | High |
| TCGA-B0-5096 | 8 | 7 | 7 | 6 | Low |
| TCGA-B0-5097 | 7 | 7 | 7 | 7 | High |
| TCGA-B0-5098 | 10 | 8 | 9 | 7 | High |
| TCGA-B0-5099 | 8 | 7 | 7 | 6 | Low |
| TCGA-B0-5100 | 9 | 7 | 8 | 6 | High |
| TCGA-B0-5102 | 8 | 7 | 7 | 6 | High |
| TCGA-B0-5106 | 9 | 8 | 8 | 7 | High |
| TCGA-B0-5107 | 9 | 9 | 9 | 9 | High |
| TCGA-B0-5108 | 7 | 7 | 6 | 7 | High |
| TCGA-B0-5109 | 7 | 7 | 7 | 7 | High |
| TCGA-B0-5110 | 8 | 8 | 8 | 7 | Low |
| TCGA-B0-5113 | 8 | 8 | 8 | 8 | Low |
| TCGA-B0-5115 | 9 | 8 | 8 | 7 | Low |
| TCGA-B0-5116 | 9 | 7 | 8 | 6 | High |
| TCGA-B0-5117 | 10 | 8 | 9 | 8 | Low |
| TCGA-B0-5119 | 8 | 8 | 8 | 7 | Low |
| TCGA-B0-5120 | 8 | 7 | 8 | 7 | Low |
| TCGA-B0-5121 | 10 | 9 | 9 | 8 | High |
| TCGA-B0-5399 | 10 | 8 | 9 | 8 | Low |
| TCGA-B0-5400 | 9 | 8 | 9 | 8 | High |
| TCGA-B0-5402 | 8 | 7 | 7 | 7 | Low |
| TCGA-B0-5690 | 8 | 7 | 8 | 6 | Low |
| TCGA-B0-5691 | 8 | 7 | 7 | 6 | Low |
| TCGA-B0-5692 | 10 | 10 | 9 | 9 | High |
| TCGA-B0-5693 | 8 | 7 | 8 | 6 | Low |
| TCGA-B0-5694 | 8 | 6 | 7 | 6 | Low |
| TCGA-B0-5695 | 8 | 7 | 8 | 7 | Low |
| TCGA-B0-5696 | 9 | 8 | 8 | 7 | High |
| TCGA-B0-5697 | 9 | 9 | 9 | 8 | High |
| TCGA-B0-5698 | 8 | 8 | 8 | 7 | High |
| TCGA-B0-5699 | 10 | 8 | 9 | 8 | Low |
| TCGA-B0-5700 | 9 | 8 | 9 | 7 | High |
| TCGA-B0-5701 | 9 | 8 | 8 | 7 | Low |
| TCGA-B0-5702 | 8 | 6 | 7 | 6 | Low |
| TCGA-B0-5703 | 9 | 8 | 9 | 7 | High |
| TCGA-B0-5705 | 10 | 9 | 9 | 9 | Low |
| TCGA-B0-5706 | 9 | 9 | 9 | 9 | High |
| TCGA-B0-5707 | 9 | 7 | 8 | 7 | High |
| TCGA-B0-5709 | 9 | 9 | 8 | 8 | Low |
| TCGA-B0-5710 | 9 | 8 | 8 | 7 | Low |
| TCGA-B0-5711 | 8 | 8 | 8 | 7 | Low |
| TCGA-B0-5712 | 10 | 9 | 9 | 8 | Low |
| TCGA-B0-5713 | 9 | 8 | 8 | 8 | Low |
| TCGA-B0-5812 | 8 | 7 | 7 | 6 | Low |
| TCGA-B2-3923 | 9 | 7 | 8 | 7 | Low |
| TCGA-B2-3924 | 9 | 10 | 9 | 10 | High |
| TCGA-B2-4098 | 7 | 7 | 7 | 7 | Low |
| TCGA-B2-4099 | 9 | 8 | 9 | 8 | High |
| TCGA-B2-4101 | 9 | 8 | 8 | 8 | Low |
| TCGA-B2-4102 | 8 | 7 | 8 | 7 | Low |
| TCGA-B2-5633 | 8 | 7 | 8 | 7 | High |
| TCGA-B2-5635 | 9 | 9 | 9 | 8 | Low |
| TCGA-B2-5636 | 8 | 7 | 7 | 6 | Low |
| TCGA-B2-5639 | 9 | 8 | 9 | 8 | Low |
| TCGA-B2-5641 | 9 | 9 | 9 | 9 | Low |
| TCGA-B2-A4SR | 8 | 8 | 8 | 7 | High |
| TCGA-B4-5377 | 9 | 8 | 8 | 8 | Low |
| TCGA-B4-5378 | 8 | 7 | 8 | 6 | Low |
| TCGA-B4-5832 | 8 | 7 | 8 | 7 | Low |
| TCGA-B4-5834 | 9 | 8 | 8 | 7 | Low |
| TCGA-B4-5835 | 9 | 8 | 9 | 7 | Low |
| TCGA-B4-5836 | 9 | 8 | 8 | 7 | Low |
| TCGA-B4-5838 | 7 | 7 | 7 | 6 | Low |
| TCGA-B4-5843 | 8 | 7 | 7 | 6 | Low |
| TCGA-B4-5844 | 8 | 7 | 7 | 6 | Low |
| TCGA-B8-4143 | 8 | 9 | 9 | 9 | High |
| TCGA-B8-4146 | 8 | 7 | 8 | 7 | Low |
| TCGA-B8-4148 | 9 | 10 | 9 | 10 | High |
| TCGA-B8-4151 | 9 | 8 | 8 | 7 | Low |
| TCGA-B8-4153 | 9 | 8 | 8 | 7 | Low |
| TCGA-B8-4154 | 9 | 8 | 9 | 8 | Low |
| TCGA-B8-4619 | 9 | 8 | 8 | 7 | Low |
| TCGA-B8-4620 | 7 | 6 | 7 | 6 | High |
| TCGA-B8-4621 | 8 | 7 | 8 | 7 | Low |
| TCGA-B8-4622 | 8 | 7 | 8 | 7 | High |
| TCGA-B8-5158 | 8 | 7 | 7 | 6 | High |
| TCGA-B8-5159 | 9 | 8 | 8 | 8 | Low |
| TCGA-B8-5162 | 9 | 9 | 8 | 9 | High |
| TCGA-B8-5163 | 8 | 9 | 8 | 8 | High |
| TCGA-B8-5164 | 9 | 10 | 9 | 9 | Low |
| TCGA-B8-5165 | 8 | 8 | 8 | 7 | Low |
| TCGA-B8-5545 | 8 | 7 | 7 | 7 | High |
| TCGA-B8-5546 | 9 | 7 | 8 | 6 | Low |
| TCGA-B8-5549 | 8 | 8 | 8 | 8 | Low |
| TCGA-B8-5550 | 8 | 7 | 7 | 7 | Low |
| TCGA-B8-5551 | 7 | 8 | 7 | 8 | High |
| TCGA-B8-5552 | 8 | 7 | 7 | 7 | Low |
| TCGA-B8-5553 | 9 | 8 | 8 | 8 | Low |
| TCGA-B8-A54D | 10 | 9 | 10 | 9 | High |
| TCGA-B8-A54E | 8 | 7 | 8 | 7 | Low |
| TCGA-B8-A54F | 8 | 6 | 7 | 6 | High |
| TCGA-B8-A54G | 10 | 10 | 10 | 10 | High |
| TCGA-B8-A54H | 9 | 8 | 8 | 7 | High |
| TCGA-B8-A54I | 8 | 8 | 8 | 7 | High |
| TCGA-B8-A54J | 9 | 8 | 9 | 8 | High |
| TCGA-B8-A54K | 8 | 6 | 7 | 6 | Low |
| TCGA-B8-A7U6 | 9 | 7 | 8 | 7 | High |
| TCGA-B8-A8YJ | 8 | 8 | 8 | 7 | High |
| TCGA-BP-4158 | 9 | 8 | 9 | 7 | High |
| TCGA-BP-4159 | 9 | 7 | 8 | 7 | Low |
| TCGA-BP-4160 | 9 | 9 | 9 | 9 | High |
| TCGA-BP-4161 | 8 | 8 | 8 | 8 | Low |
| TCGA-BP-4162 | 8 | 9 | 8 | 9 | Low |
| TCGA-BP-4163 | 8 | 7 | 7 | 6 | Low |
| TCGA-BP-4164 | 8 | 7 | 8 | 7 | Low |
| TCGA-BP-4165 | 10 | 9 | 9 | 8 | High |
| TCGA-BP-4166 | 9 | 8 | 8 | 7 | Low |
| TCGA-BP-4167 | 8 | 8 | 8 | 8 | High |
| TCGA-BP-4169 | 8 | 7 | 8 | 7 | High |
| TCGA-BP-4170 | 9 | 9 | 8 | 8 | Low |
| TCGA-BP-4173 | 8 | 9 | 9 | 9 | High |
| TCGA-BP-4174 | 9 | 8 | 8 | 8 | Low |
| TCGA-BP-4176 | 8 | 7 | 8 | 7 | High |
| TCGA-BP-4177 | 9 | 7 | 8 | 6 | Low |
| TCGA-BP-4325 | 10 | 10 | 9 | 9 | High |
| TCGA-BP-4326 | 7 | 6 | 7 | 6 | High |
| TCGA-BP-4327 | 8 | 6 | 7 | 6 | High |
| TCGA-BP-4329 | 9 | 8 | 8 | 7 | Low |
| TCGA-BP-4330 | 9 | 9 | 9 | 9 | Low |
| TCGA-BP-4331 | 8 | 7 | 8 | 7 | High |
| TCGA-BP-4332 | 8 | 6 | 7 | 6 | High |
| TCGA-BP-4334 | 10 | 8 | 9 | 7 | Low |
| TCGA-BP-4335 | 9 | 9 | 9 | 8 | High |
| TCGA-BP-4337 | 8 | 7 | 7 | 6 | High |
| TCGA-BP-4338 | 8 | 7 | 7 | 6 | Low |
| TCGA-BP-4340 | 8 | 7 | 8 | 7 | Low |
| TCGA-BP-4341 | 8 | 7 | 8 | 6 | High |
| TCGA-BP-4342 | 8 | 7 | 8 | 7 | High |
| TCGA-BP-4343 | 8 | 8 | 8 | 7 | Low |
| TCGA-BP-4344 | 8 | 7 | 8 | 7 | Low |
| TCGA-BP-4345 | 8 | 8 | 7 | 7 | High |
| TCGA-BP-4346 | 9 | 10 | 9 | 10 | High |
| TCGA-BP-4347 | 9 | 8 | 9 | 8 | Low |
| TCGA-BP-4349 | 9 | 7 | 8 | 7 | High |
| TCGA-BP-4351 | 9 | 7 | 8 | 7 | High |
| TCGA-BP-4352 | 8 | 7 | 7 | 6 | High |
| TCGA-BP-4353 | 8 | 8 | 8 | 7 | High |
| TCGA-BP-4354 | 7 | 7 | 6 | 6 | High |
| TCGA-BP-4355 | 8 | 6 | 7 | 6 | High |
| TCGA-BP-4756 | 8 | 7 | 7 | 7 | Low |
| TCGA-BP-4758 | 10 | 9 | 9 | 9 | High |
| TCGA-BP-4759 | 9 | 8 | 8 | 7 | Low |
| TCGA-BP-4760 | 7 | 6 | 7 | 6 | Low |
| TCGA-BP-4761 | 8 | 7 | 8 | 7 | High |
| TCGA-BP-4762 | 9 | 8 | 8 | 7 | Low |
| TCGA-BP-4763 | 9 | 8 | 8 | 7 | Low |
| TCGA-BP-4765 | 8 | 7 | 8 | 6 | Low |
| TCGA-BP-4766 | 8 | 6 | 7 | 6 | Low |
| TCGA-BP-4768 | 8 | 7 | 7 | 6 | Low |
| TCGA-BP-4769 | 9 | 7 | 8 | 6 | Low |
| TCGA-BP-4770 | 7 | 6 | 7 | 6 | High |
| TCGA-BP-4771 | 9 | 10 | 9 | 10 | High |
| TCGA-BP-4774 | 9 | 8 | 8 | 7 | Low |
| TCGA-BP-4775 | 8 | 7 | 7 | 7 | Low |
| TCGA-BP-4776 | 9 | 8 | 9 | 8 | High |
| TCGA-BP-4777 | 9 | 9 | 9 | 9 | High |
| TCGA-BP-4781 | 8 | 7 | 7 | 6 | Low |
| TCGA-BP-4782 | 9 | 8 | 9 | 8 | High |
| TCGA-BP-4784 | 9 | 7 | 8 | 6 | Low |
| TCGA-BP-4787 | 8 | 7 | 7 | 6 | High |
| TCGA-BP-4789 | 9 | 7 | 8 | 7 | Low |
| TCGA-BP-4790 | 8 | 8 | 8 | 8 | High |
| TCGA-BP-4795 | 8 | 7 | 7 | 6 | Low |
| TCGA-BP-4797 | 9 | 8 | 8 | 7 | Low |
| TCGA-BP-4798 | 9 | 10 | 9 | 9 | High |
| TCGA-BP-4799 | 9 | 9 | 9 | 8 | Low |
| TCGA-BP-4801 | 8 | 7 | 7 | 7 | Low |
| TCGA-BP-4803 | 9 | 7 | 8 | 6 | Low |
| TCGA-BP-4804 | 9 | 9 | 9 | 9 | High |
| TCGA-BP-4807 | 7 | 6 | 6 | 5 | Low |
| TCGA-BP-4959 | 8 | 7 | 8 | 7 | Low |
| TCGA-BP-4960 | 9 | 7 | 8 | 7 | High |
| TCGA-BP-4961 | 9 | 7 | 8 | 7 | Low |
| TCGA-BP-4962 | 9 | 8 | 8 | 8 | High |
| TCGA-BP-4963 | 8 | 7 | 8 | 7 | High |
| TCGA-BP-4964 | 9 | 8 | 8 | 7 | High |
| TCGA-BP-4965 | 9 | 8 | 8 | 7 | Low |
| TCGA-BP-4967 | 8 | 7 | 8 | 6 | Low |
| TCGA-BP-4968 | 9 | 8 | 9 | 8 | High |
| TCGA-BP-4969 | 9 | 7 | 8 | 7 | Low |
| TCGA-BP-4970 | 9 | 9 | 8 | 8 | Low |
| TCGA-BP-4971 | 9 | 8 | 9 | 8 | High |
| TCGA-BP-4972 | 7 | 7 | 7 | 6 | Low |
| TCGA-BP-4973 | 8 | 8 | 8 | 8 | Low |
| TCGA-BP-4974 | 9 | 7 | 8 | 7 | High |
| TCGA-BP-4975 | 8 | 6 | 7 | 6 | Low |
| TCGA-BP-4976 | 8 | 7 | 7 | 6 | Low |
| TCGA-BP-4977 | 9 | 8 | 8 | 7 | Low |
| TCGA-BP-4981 | 10 | 9 | 9 | 8 | High |
| TCGA-BP-4982 | 9 | 8 | 8 | 7 | Low |
| TCGA-BP-4983 | 6 | 8 | 6 | 8 | Low |
| TCGA-BP-4985 | 8 | 8 | 8 | 7 | High |
| TCGA-BP-4986 | 8 | 7 | 8 | 7 | Low |
| TCGA-BP-4987 | 9 | 8 | 8 | 7 | Low |
| TCGA-BP-4989 | 9 | 9 | 9 | 9 | High |
| TCGA-BP-4991 | 8 | 7 | 8 | 7 | High |
| TCGA-BP-4992 | 9 | 8 | 9 | 9 | High |
| TCGA-BP-4993 | 10 | 9 | 9 | 8 | High |
| TCGA-BP-4994 | 9 | 8 | 8 | 7 | Low |
| TCGA-BP-4995 | 9 | 8 | 8 | 7 | Low |
| TCGA-BP-4998 | 8 | 8 | 8 | 7 | High |
| TCGA-BP-4999 | 9 | 8 | 8 | 7 | Low |
| TCGA-BP-5000 | 9 | 8 | 9 | 8 | High |
| TCGA-BP-5001 | 9 | 8 | 9 | 7 | High |
| TCGA-BP-5004 | 9 | 8 | 8 | 7 | Low |
| TCGA-BP-5006 | 9 | 8 | 8 | 7 | Low |
| TCGA-BP-5007 | 9 | 8 | 8 | 8 | High |
| TCGA-BP-5008 | 9 | 8 | 8 | 8 | High |
| TCGA-BP-5009 | 8 | 7 | 8 | 7 | High |
| TCGA-BP-5010 | 9 | 7 | 9 | 7 | High |
| TCGA-BP-5168 | 9 | 8 | 8 | 7 | High |
| TCGA-BP-5169 | 9 | 8 | 9 | 8 | High |
| TCGA-BP-5170 | 8 | 7 | 8 | 6 | Low |
| TCGA-BP-5173 | 10 | 9 | 9 | 9 | Low |
| TCGA-BP-5174 | 9 | 8 | 9 | 7 | High |
| TCGA-BP-5175 | 10 | 8 | 9 | 8 | High |
| TCGA-BP-5176 | 10 | 8 | 9 | 8 | High |
| TCGA-BP-5177 | 9 | 8 | 9 | 7 | Low |
| TCGA-BP-5178 | 9 | 8 | 9 | 8 | High |
| TCGA-BP-5180 | 9 | 7 | 8 | 7 | High |
| TCGA-BP-5181 | 9 | 8 | 8 | 7 | High |
| TCGA-BP-5182 | 8 | 8 | 8 | 7 | Low |
| TCGA-BP-5183 | 10 | 9 | 9 | 8 | Low |
| TCGA-BP-5184 | 9 | 8 | 8 | 7 | Low |
| TCGA-BP-5185 | 9 | 8 | 9 | 7 | High |
| TCGA-BP-5186 | 9 | 8 | 8 | 8 | Low |
| TCGA-BP-5187 | 9 | 8 | 9 | 8 | Low |
| TCGA-BP-5189 | 8 | 7 | 8 | 7 | Low |
| TCGA-BP-5190 | 10 | 9 | 10 | 8 | Low |
| TCGA-BP-5191 | 10 | 9 | 10 | 9 | High |
| TCGA-BP-5192 | 9 | 7 | 8 | 7 | Low |
| TCGA-BP-5194 | 8 | 7 | 8 | 7 | Low |
| TCGA-BP-5195 | 9 | 8 | 8 | 7 | Low |
| TCGA-BP-5196 | 9 | 9 | 9 | 8 | High |
| TCGA-BP-5198 | 8 | 7 | 7 | 6 | High |
| TCGA-BP-5199 | 9 | 9 | 9 | 9 | High |
| TCGA-BP-5200 | 8 | 8 | 8 | 8 | High |
| TCGA-BP-5201 | 10 | 9 | 10 | 9 | High |
| TCGA-BP-5202 | 9 | 8 | 8 | 7 | Low |
| TCGA-CJ-4634 | 8 | 7 | 7 | 6 | Low |
| TCGA-CJ-4635 | 8 | 8 | 8 | 8 | High |
| TCGA-CJ-4636 | 9 | 8 | 8 | 8 | High |
| TCGA-CJ-4637 | 8 | 9 | 9 | 9 | High |
| TCGA-CJ-4638 | 10 | 8 | 10 | 8 | Low |
| TCGA-CJ-4639 | 9 | 9 | 8 | 8 | Low |
| TCGA-CJ-4640 | 10 | 10 | 9 | 9 | Low |
| TCGA-CJ-4641 | 9 | 9 | 9 | 9 | Low |
| TCGA-CJ-4642 | 8 | 7 | 8 | 6 | High |
| TCGA-CJ-4643 | 8 | 8 | 8 | 7 | Low |
| TCGA-CJ-4644 | 8 | 8 | 7 | 7 | Low |
| TCGA-CJ-4868 | 8 | 8 | 8 | 8 | High |
| TCGA-CJ-4869 | 10 | 10 | 10 | 10 | High |
| TCGA-CJ-4870 | 8 | 7 | 8 | 7 | High |
| TCGA-CJ-4871 | 9 | 8 | 8 | 7 | High |
| TCGA-CJ-4872 | 9 | 8 | 9 | 8 | High |
| TCGA-CJ-4873 | 9 | 8 | 9 | 8 | High |
| TCGA-CJ-4874 | 9 | 9 | 9 | 8 | Low |
| TCGA-CJ-4875 | 9 | 8 | 8 | 8 | High |
| TCGA-CJ-4876 | 10 | 9 | 9 | 8 | High |
| TCGA-CJ-4878 | 8 | 7 | 8 | 7 | High |
| TCGA-CJ-4881 | 9 | 7 | 8 | 7 | High |
| TCGA-CJ-4882 | 9 | 8 | 9 | 8 | High |
| TCGA-CJ-4884 | 8 | 8 | 8 | 7 | Low |
| TCGA-CJ-4885 | 9 | 7 | 8 | 7 | Low |
| TCGA-CJ-4886 | 9 | 8 | 8 | 8 | Low |
| TCGA-CJ-4887 | 10 | 9 | 9 | 9 | High |
| TCGA-CJ-4888 | 8 | 9 | 8 | 9 | High |
| TCGA-CJ-4889 | 9 | 9 | 9 | 8 | High |
| TCGA-CJ-4890 | 8 | 9 | 8 | 8 | High |
| TCGA-CJ-4891 | 9 | 7 | 8 | 7 | High |
| TCGA-CJ-4892 | 9 | 9 | 9 | 8 | High |
| TCGA-CJ-4893 | 8 | 8 | 8 | 8 | Low |
| TCGA-CJ-4894 | 9 | 9 | 9 | 9 | High |
| TCGA-CJ-4895 | 8 | 7 | 8 | 7 | High |
| TCGA-CJ-4897 | 9 | 8 | 8 | 7 | Low |
| TCGA-CJ-4899 | 8 | 8 | 8 | 7 | High |
| TCGA-CJ-4900 | 9 | 8 | 9 | 8 | High |
| TCGA-CJ-4901 | 8 | 9 | 8 | 8 | High |
| TCGA-CJ-4902 | 8 | 8 | 8 | 7 | High |
| TCGA-CJ-4903 | 8 | 8 | 8 | 7 | Low |
| TCGA-CJ-4904 | 8 | 8 | 8 | 7 | High |
| TCGA-CJ-4905 | 9 | 7 | 8 | 7 | Low |
| TCGA-CJ-4907 | 8 | 7 | 8 | 6 | Low |
| TCGA-CJ-4908 | 9 | 8 | 8 | 8 | High |
| TCGA-CJ-4912 | 8 | 7 | 7 | 6 | High |
| TCGA-CJ-4916 | 9 | 9 | 9 | 9 | High |
| TCGA-CJ-4918 | 8 | 7 | 7 | 7 | Low |
| TCGA-CJ-4920 | 8 | 7 | 7 | 6 | High |
| TCGA-CJ-5671 | 8 | 7 | 7 | 7 | High |
| TCGA-CJ-5672 | 9 | 9 | 9 | 9 | High |
| TCGA-CJ-5675 | 9 | 10 | 9 | 9 | High |
| TCGA-CJ-5676 | 9 | 8 | 8 | 7 | High |
| TCGA-CJ-5677 | 9 | 7 | 8 | 6 | High |
| TCGA-CJ-5678 | 10 | 9 | 10 | 9 | Low |
| TCGA-CJ-5679 | 9 | 7 | 8 | 6 | High |
| TCGA-CJ-5680 | 8 | 7 | 7 | 6 | Low |
| TCGA-CJ-5681 | 8 | 6 | 7 | 6 | Low |
| TCGA-CJ-5682 | 9 | 8 | 8 | 7 | High |
| TCGA-CJ-5683 | 9 | 8 | 9 | 7 | Low |
| TCGA-CJ-5684 | 10 | 9 | 9 | 8 | Low |
| TCGA-CJ-5686 | 8 | 8 | 8 | 7 | Low |
| TCGA-CJ-6027 | 8 | 8 | 8 | 8 | High |
| TCGA-CJ-6028 | 8 | 8 | 8 | 8 | Low |
| TCGA-CJ-6030 | 8 | 7 | 8 | 7 | High |
| TCGA-CJ-6031 | 9 | 8 | 8 | 7 | High |
| TCGA-CJ-6032 | 8 | 8 | 8 | 7 | High |
| TCGA-CJ-6033 | 8 | 7 | 8 | 6 | High |
| TCGA-CW-5580 | 7 | 6 | 7 | 6 | Low |
| TCGA-CW-5581 | 9 | 8 | 8 | 7 | Low |
| TCGA-CW-5583 | 8 | 7 | 7 | 6 | Low |
| TCGA-CW-5584 | 8 | 7 | 8 | 6 | Low |
| TCGA-CW-5585 | 8 | 6 | 7 | 5 | Low |
| TCGA-CW-5587 | 8 | 8 | 8 | 8 | Low |
| TCGA-CW-5588 | 8 | 7 | 7 | 6 | High |
| TCGA-CW-5589 | 8 | 7 | 7 | 6 | Low |
| TCGA-CW-5590 | 9 | 8 | 8 | 7 | High |
| TCGA-CW-5591 | 7 | 6 | 7 | 5 | Low |
| TCGA-CW-6087 | 7 | 9 | 7 | 9 | High |
| TCGA-CW-6088 | 8 | 7 | 8 | 7 | Low |
| TCGA-CW-6090 | 7 | 7 | 7 | 6 | Low |
| TCGA-CW-6093 | 8 | 7 | 7 | 6 | Low |
| TCGA-CW-6097 | 8 | 8 | 8 | 8 | Low |
| TCGA-CZ-4853 | 9 | 8 | 8 | 7 | Low |
| TCGA-CZ-4854 | 8 | 7 | 8 | 7 | High |
| TCGA-CZ-4856 | 10 | 9 | 10 | 9 | High |
| TCGA-CZ-4857 | 8 | 7 | 7 | 7 | High |
| TCGA-CZ-4858 | 8 | 8 | 8 | 8 | High |
| TCGA-CZ-4859 | 8 | 7 | 8 | 6 | Low |
| TCGA-CZ-4860 | 8 | 7 | 7 | 6 | Low |
| TCGA-CZ-4861 | 7 | 7 | 7 | 7 | Low |
| TCGA-CZ-4862 | 9 | 9 | 9 | 9 | High |
| TCGA-CZ-4863 | 9 | 9 | 9 | 9 | High |
| TCGA-CZ-4864 | 9 | 10 | 9 | 9 | High |
| TCGA-CZ-4865 | 9 | 8 | 9 | 8 | High |
| TCGA-CZ-4866 | 8 | 7 | 8 | 7 | Low |
| TCGA-CZ-5451 | 9 | 8 | 9 | 8 | High |
| TCGA-CZ-5452 | 10 | 10 | 9 | 9 | High |
| TCGA-CZ-5453 | 8 | 7 | 8 | 7 | Low |
| TCGA-CZ-5454 | 7 | 7 | 7 | 6 | Low |
| TCGA-CZ-5455 | 9 | 9 | 9 | 9 | Low |
| TCGA-CZ-5456 | 9 | 8 | 8 | 7 | High |
| TCGA-CZ-5457 | 9 | 8 | 8 | 8 | Low |
| TCGA-CZ-5458 | 9 | 8 | 8 | 8 | Low |
| TCGA-CZ-5459 | 9 | 8 | 9 | 8 | High |
| TCGA-CZ-5460 | 9 | 8 | 8 | 8 | High |
| TCGA-CZ-5461 | 8 | 7 | 8 | 7 | High |
| TCGA-CZ-5462 | 9 | 8 | 9 | 8 | High |
| TCGA-CZ-5463 | 9 | 8 | 8 | 8 | Low |
| TCGA-CZ-5464 | 9 | 8 | 8 | 8 | High |
| TCGA-CZ-5465 | 8 | 7 | 7 | 6 | Low |
| TCGA-CZ-5466 | 9 | 8 | 8 | 8 | High |
| TCGA-CZ-5467 | 8 | 7 | 8 | 6 | Low |
| TCGA-CZ-5468 | 9 | 9 | 9 | 8 | High |
| TCGA-CZ-5469 | 9 | 7 | 8 | 7 | High |
| TCGA-CZ-5470 | 9 | 9 | 9 | 8 | Low |
| TCGA-CZ-5982 | 8 | 7 | 8 | 7 | High |
| TCGA-CZ-5984 | 9 | 7 | 8 | 7 | High |
| TCGA-CZ-5985 | 8 | 8 | 8 | 7 | Low |
| TCGA-CZ-5986 | 8 | 7 | 7 | 7 | Low |
| TCGA-CZ-5987 | 9 | 8 | 9 | 8 | High |
| TCGA-CZ-5988 | 8 | 7 | 8 | 7 | High |
| TCGA-CZ-5989 | 8 | 8 | 8 | 7 | Low |
| TCGA-DV-5565 | 8 | 7 | 8 | 7 | High |
| TCGA-DV-5566 | 10 | 8 | 9 | 8 | High |
| TCGA-DV-5567 | 8 | 6 | 7 | 5 | Low |
| TCGA-DV-5568 | 8 | 8 | 8 | 7 | High |
| TCGA-DV-5569 | 9 | 8 | 8 | 8 | High |
| TCGA-DV-5573 | 10 | 10 | 10 | 10 | High |
| TCGA-DV-5574 | 9 | 9 | 9 | 8 | High |
| TCGA-DV-5575 | 9 | 8 | 9 | 8 | High |
| TCGA-DV-5576 | 8 | 6 | 8 | 6 | Low |
| TCGA-DV-A4VX | 8 | 7 | 8 | 7 | High |
| TCGA-DV-A4VZ | 9 | 7 | 8 | 7 | High |
| TCGA-DV-A4W0 | 10 | 8 | 9 | 8 | High |
| TCGA-EU-5904 | 8 | 7 | 7 | 7 | Low |
| TCGA-EU-5905 | 8 | 7 | 8 | 6 | Low |
| TCGA-EU-5906 | 8 | 7 | 7 | 6 | Low |
| TCGA-EU-5907 | 7 | 7 | 7 | 6 | High |
| TCGA-G6-A5PC | 10 | 9 | 9 | 8 | High |
| TCGA-G6-A8L6 | 9 | 8 | 8 | 7 | High |
| TCGA-G6-A8L7 | 8 | 7 | 8 | 7 | High |
| TCGA-G6-A8L8 | 9 | 7 | 8 | 7 | High |
| TCGA-GK-A6C7 | 9 | 8 | 8 | 7 | High |
| TCGA-MM-A563 | 8 | 8 | 8 | 8 | High |
| TCGA-MM-A564 | 9 | 7 | 8 | 7 | High |
| TCGA-MM-A84U | 9 | 8 | 8 | 7 | High |
| TCGA-MW-A4EC | 9 | 8 | 9 | 8 | High |
| TCGA-T7-A92I | 8 | 8 | 8 | 7 | Low |
